# Toll-like receptor 4 signaling-mediated responses are critically engaged in optimal host protection against highly virulent *Mycobacterium tuberculosis* K infection

**DOI:** 10.1080/21505594.2020.1766401

**Published:** 2020-05-13

**Authors:** Jaehun Park, Hongmin Kim, Kee Woong Kwon, Hong-Hee Choi, Soon Myung Kang, Jung Joo Hong, Sung Jae Shin

**Affiliations:** a Department of Microbiology, Institute for Immunology and Immunological Disease, Brain Korea 21 PLUS Project for Medical Science, Yonsei University College of Medicine, Seoul, South Korea; b National Primate Research Center, Korea Research Institute of Bioscience and Biotechnology, Cheongju, South Korea

**Keywords:** *Mycobacterium tuberculosis*, TLR4, neutrophil, IL-10, IL-10 receptor

## Abstract

Toll-like receptors (TLRs) play critical roles in the innate recognition of *Mycobacterium tuberculosis* (Mtb) by host immune cells. However, controversy has arisen regarding the role of TLR4 in determining the outcomes of Mtb infection. To address this controversy, the function of TLR4 in the induction of an optimal protective immune response against the highly virulent Mtb K-infection was comparatively investigated in C3 H/HeJ (TLR4-deficient mutant) and C3 H/HeN (TLR4-competent wild-type) mice. Interestingly, following Mtb infection, C3 H/HeJ mice showed a more severe disease phenotype than C3 H/HeN mice, exhibiting reduced weight and a marked increase in bacterial burden along with necrotic lung inflammation. Analysis of the immune cell composition revealed significantly increased neutrophils in the lung and significant production of IL-10 accompanied by the impairment of the protective Th1 response in C3 H/HeJ mice. Reducing the neutrophil numbers by treating C3 H/HeJ mice with an anti-Ly6 G monoclonal antibody (mAb) and blocking IL-10 signaling with an anti-IL-10 receptor mAb reduced the excessive lung inflammation and bacterial burden in C3 H/HeJ mice. Therefore, abundant IL-10 signaling and neutrophils have detrimental effects in TLR4-deficient mice during Mtb infection. However, the blockade of IL-10 signaling produced an increase in the CD11b^hi^Ly6 G^hi^ neutrophil population, but the phenotypes of these neutrophils were different from those of the CD11b^int^Ly6 G^int^ neutrophils from mice with controlled infections. Collectively, these results show that TLR4 positively contributes to the generation of an optimal protective immunity against Mtb infection. Furthermore, investigating the TLR4-mediated response will provide insight for the development of effective control measures against tuberculosis.

## Introduction

Tuberculosis (TB), caused by *Mycobacterium tuberculosis* (Mtb), is highly contagious and the leading infectious disease, causing 1.6 million human deaths worldwide in 2017 [1]. TB is a life-threatening disease that is newly diagnosed more than 10 million times every year. The design of an effective treatment and vaccine for TB control by dissecting various virulence factors requires investigations of the primary immune responses that protect the host and the immunological understanding of the susceptibility to TB. The incomplete understanding of the pathogenesis of Mtb infection makes it difficult to control this detrimental pathogen.

The innate recognition of mycobacterial products is the first step in a series of events leading to effective host defense against Mtb infection. Antigen-presenting cells, such as macrophages and dendritic cells, express pattern recognition receptors (PRRs) that recognize conserved molecular patterns, the so-called pathogen-associated molecular patterns (PAMPs). Toll-like receptors (TLRs) are one of the well-characterized PRR families. Functionally, TLRs 1–10 in humans and TLRs 1–9 and 11–13 in mice have been discovered; consequently, studies of the immune response related to various bacteria, viruses and fungi are underway. Among the TLRs, TLR2, TLR4, and TLR9 are well known to be involved in the recognition of Mtb [2]. In addition, genetic risk for Mtb infection may be increased by defects or polymorphisms in the TLR family. Polymorphisms in *TLR2* and *TLR4* genes might cause a reduced macrophage response to bacterial components, resulting in increased susceptibility to TB [3].

Appropriate immune responses mediated by host cells present resistance to TB. In particular, interferon-gamma (IFN)-γ-secreting CD4^+^ T cells are essential for the protective immune response to mycobacterial infection [4]. Various animal models with a disrupted IFN-γ response are unable to restrain the growth of Mtb and capitulate to the infection [5,6]. Other types of inflammatory cytokines, such as tumor necrosis factor (TNF)-α and interleukin (IL)-12, are important for limiting Mtb infection through granuloma formation and activating T cell responses [7]. In contrast to pro-inflammatory cytokines such as TNF-α and IL-12, anti-inflammatory cytokines such as IL-10 inhibit the immune response of the host, creating a favorable environment for the growth of Mtb. A number of studies have shown that IL-10 secreted in response to Mtb is associated with susceptibility to TB in human and mouse models [8]. The ablation of IL-10 signaling in mice infected with Mtb is beneficial for the control of bacterial growth and improves mouse survival due to the restoration of CD4^+^ T cells and the T helper (Th) 1 responses [9].

Although several antigens derived from Mtb or Mtb itself bind to TLR4 [10], resulting in a variety of alterations in immunobiological responses such as immune activation or cell death promotion, there remains controversy around the functional role TLR4 plays in the pathogenesis of TB. C3 H/HeJ and C3 H/HeN mouse substrains were derived from the same parental strain C3 H/He in 1947 [11]. C3 H/HeJ mice carry a missense mutation in the *TLR4* gene, which induces a single amino acid change in the cytoplasmic portion of TLR4, disrupts signal transduction and induces a phenotype similar to that of TLR4-knockout mice [12]. C3 H/HeJ mice have a specific tolerance to lipopolysaccharide (LPS), unlike C3 H/HeN, due to the TLR4 mutation [12]. However, C3 H/HeJ mice are highly susceptible to infection by bacteria, such as *Escherichia coli* and *Salmonella enterica* [13,14].

Components of Mtb, such as 3- and 4-acylated lipomannan and 60- and 65-heat shock proteins, activate various immune cell pathways when they bind to TLR4 [15]. In particular, TLR4 ligation plays a major role in inducing Th1 responses by activating dendritic cells, which interact with T cells [16]. Because of this characteristic, TLR4 agonists have been developed as vaccine adjuvant candidates for influenza, polio, and hepatitis [17]. Adjuvants from TB are being studied using glucopyranosyl lipid adjuvant (GLA), which is a TLR4 agonist combined with a stable emulsion formulation, to increase the efficacy of the existing vaccine [18]. However, the role of TLR4 during Mtb infection is still under discussion. Previous studies have shown inconsistent results regarding the protective role TLR4 plays against Mtb infection. For example, Abel *et al*. reported that TLR4 is necessary to control chronic Mtb infection in mice, indicating that TLR4 mediates the protective immune response during chronic TB [19]. In addition, Branger *et al*. showed that TLR4 is required to produce IFN-γ upon antigen-specific stimulation, suggesting that TLR4 plays a protective role in host defense against lung infection by Mtb [20]. However, other studies have demonstrated that TLR4 does not protect against Mtb infection. For example, Shim *et al*. showed that TLR4-competent and TLR4-deficient mice showed similar types of cell accumulation and lung histopathology [21]. Furthermore, Reiling *et al*. showed that TLR4 signaling is not required to control Mtb growth [22].

In this study, we challenged C57BL/6 J, C3 H/HeN, and C3 H/HeJ mice to clarify the functional role of TLR4 and to define the immunological alterations that are involved in host protection during clinical Mtb infection. High levels of IL-10 secretion and neutrophil recruitment were found in the absence of TLR4 in mice. These results indicate that TLR4 might be involved in susceptibility to TB. Thus, our research reveals that TLR4 could be necessary for host protection against pulmonary TB.

## Materials and Methods

### Experimental animals and ethics statement

Specific pathogen-free 6- or 7-week-old C57BL/6 J, C3 H/HeN, and C3 H/HeJ mice were purchased from Japan SLC, Inc. (Shijuoka, Japan) or the Jackson Laboratory (Bar Harbor, ME, USA) and strictly maintained under barrier conditions in a Biosafety Level 3 (BSL-3) facility at Avison Biomedical Research Center at Yonsei University College of Medicine (Seoul, Korea). All animal studies were performed in accordance with the Korean Food and Drug Administration (KFDA) guidelines. The experimental protocols used in this study were approved by the Ethics Committee and Institutional Animal Care and Use Committee of the Laboratory Animal Research Center at Yonsei University College of Medicine (permit number: 2017–0342).

### Mtb K strain and culture conditions

The Mtb K strain was collected from the Korean Tuberculosis Research Institute (KIT, Osong, Chungcheongbuk-do, Korea) and was cultured for infection experiments as described previously [23].

### Mtb K strain infections and antibody treatments

Mice were infected with the Mtb K strain via aerosolization as previously described [24]. Briefly, mice were exposed to the Mtb K strain in the calibrated inhalation chamber of an airborne infection apparatus for 60 min delivering a predetermined dose (Glas-Col, Terre Haute, IN, USA); approximately 150 viable bacteria were delivered to the lungs. After 2 weeks, 250 μg/mouse anti-IL-10 receptor mAb (1B1.3A; Bio X Cell, West Lebanon, NH, USA) in PBS for the blockade of IL-10 signaling, and 250 µg/mouse anti-Ly6 G mAb (1A8; Bio X Cell, West Lebanon, NH, USA) in PBS to reduce the neutrophil number, were injected intraperitoneally 3 times per week. All mice were sacrificed 3 or 4 weeks after Mtb K strain infection.

### Antibodies and reagents

A LIVE/DEAD® Fixable Near-IR Dead Cell Stain Kit; Green Dead Cell Stain Kit; and Aqua Dead Cell Stain Kit were purchased from Molecular Probes (Carlsbad, CA, USA). A phycoerythrin (PE)-conjugated mAb against IFN-γ; violet (V) 450-conjugated mAb against CD44; brilliant violet (BV) 605-conjugated mAb against Thy1.2; PerCP-Cy5.5-conjugated mAb against CD4; ΒV 421-conjugated mAb against CD45; and a V 450-conjugated mAb against Ly6 G were purchased from BD Bioscience (San Jose, CA, USA), and peridinin chlorophyll (PerCP)-Cy5.5-conjugated mAb against CD11b, allophycocyanin (APC)-Cy7-conjugated mAb against I-Ab, PE-conjugated mAb against CD64, and PE-Dazzle-conjugated mAb against CD11 c were purchased from Biolegend (San Diego, CA, USA) for flow cytometry analyses. Recombinant mouse granulocyte-macrophage colony-stimulating factor (GM-CSF) and IL-4 were purchased from JW CreaGene (Daegu, Korea).

### Generation of bone marrow-derived dendritic cells

Red blood cells (RBCs) among whole bone marrow cells from C3 H/HeJ and C3 H/HeN mice were lysed using RBC Lysis Buffer (Sigma-Aldrich, St. Louis, MO, USA) and then the cells were maintained in Petri dishes (1 × 10^6^ cells/mL; 10 mL/Petri dish) and cultured at 37°C in the presence of 5% CO_2_ using complete-RPMI 1640 (c-RPMI 1640) supplemented with 100 U/mL penicillin/streptomycin (Lonza, Basel, Switzerland), 10% fetal bovine serum (Lonza, Basel, Switzerland), GM-CSF (20 ng/mL) and IL-4 (0.5 ng/mL). The culture plates were supplemented with 10 mL of c-RPMI 1640 medium per plate on day 3, which was replaced with 10 mL c-RPMI 1640 medium on day 6 of culture. After 9 days of culture, the cells were harvested and used for experiments.

### Preparation of single cells and immune cell analysis

Lung single-cell suspensions were prepared from mice 4 weeks after Mtb infection. To obtain a single-cell suspension, the lung tissue was minced into 2–4 mm pieces with scissors. The lung tissue was incubated in 3 mL of cellular dissociation buffer RPMI medium (Biowest, Nuaillé, France) containing 0.1% collagenase type IV (Worthington Biochemical Corporation, Lakewood, NJ, USA), 1 mM CaCl_2_ and 1 mM MgCl_2_ for 30 min at 37°C. Lung and spleen cells were separated through 40-μm cell strainers (Corning, NY, USA), and RBCs were lysed with ACK lysis buffer (Lonza, Basel, Switzerland) for 3 min at room temperature. After the cells were with c-RPMI 1640, a single-cell suspension was prepared.

### Cytokine measurement by ELISA

Single cells from the lungs and spleens of Mtb-infected or immunized mice were stimulated with early secreted antigenic targets of 6 kDa (ESAT-6) for 12 hours at 37 °C. A sandwich enzyme-linked immunosorbent assay (ELISA) was used to detect TNF-α, IFN-γ, IL-6, IL-1β, IL-12p70, IL-10, and IL-17AF (Invitrogen, San Diego, CA, USA).

### Analysis of IFN-γ secreting CD4^+^ T cells by intracellular staining

Lung single-cell suspensions were prepared from immunized and Mtb-infected mice and stimulated with 1 μg/mL ESAT-6 at 37**°C** for 12 hours in the presence of GolgiPlug and GolgiStop (BD Bioscience, San Jose, CA, USA). First, the cells were washed with PBS, and the Fc receptor was blocked with an anti-CD16/32 blocking antibody at 4 °C for 15 min. Surface molecules were stained with fluorochrome-conjugated antibodies against Thy1.2, CD4, CD44, and a Live and DEAD^TM^ Fixable Dead Cell kit for 30 min at 4 °C. After being washed with PBS, the cells were fixed and permeabilized with Cytofix/Cytoperm (BD Biosciences, San Jose, CA, USA) for 30 min at 4 °C. The permeabilized cells were washed twice with Perm/Wash (BD Biosciences, San Jose, CA, USA) and stained with PE-conjugated anti-IFN-γ, for 30 min at 4 °C. The cells were washed twice with Perm/Wash and fixed with intracellular (IC) fixation buffer (eBioscience, San Diego, CA, USA) until analysis by flow cytometry (Beckman-Coulter, Pasadena, CA, USA).

### T cell suppression assay

Ly6 G^+^ cells were obtained by anti-Ly6 G antibody via MACS system (Miltenyi Biotec, Bergisch Gladbach, Germany) from lung cells of C3 H/HeJ-infected mice. T cells were isolated from the spleens of C3 H/HeJ by microbead-conjugated anti-CD90.2 antibody via a MACS system and were labeled using a CellTrace^TM^ Violet Cell Proliferation kit (ThermoFisher Scientific, Waltham, MA, USA). After labeling, splenocytes stimulated with anti-CD3/28 (Clone: 145–2C11/37.51, BD Pharmingen) were co-cultured with varying ratios. After 72 hours of co-culture, proliferation was measured by flow cytometry.

### Histopathological analysis and enumeration of Mtb

The disease severity and phenotype were evaluated through histopathology and bacterial growth in the lungs and spleen. The organs were removed to determine protection at 4 weeks after infection. For the lung histopathology, the right-superior lobes were preserved overnight in 10% formalin and embedded in paraffin. The lungs were sectioned at 4–5 μm and H&E stained. The severity of lung inflammation was examined using the ImageJ program (National Institutes of Health, USA) as previously described [25]. For bacterial growth analysis, the lungs and spleens were homogenized, and serially diluted samples were plated onto Middlebrook 7H10 agar (Becton Dickinson, Franklin Lakes, NJ, USA) supplemented with 10% OADC (Difco Laboratories, Detroit, MI, USA), 2 μg/mL 2-thiophenecarboxylic acid hydrazide (Sigma-Aldrich, St. Louis, MO, USA) and amphotericin B (Sigma-Aldrich, St. Louis, MO, USA). After 4 weeks of incubation at 37 °C, bacterial colonies were counted.

### Statistical analyses

The results are expressed as the mean ± standard deviation (SD). The significance of differences between groups was analyzed by unpaired *t*-test in the selected two groups using GraphPad Prism version 7.0.0 for Windows (GraphPad Software, La Jolla, CA, USA, www.graphpad.com). Comparisons between two groups were conducted by an unpaired *t*-test. Statistical significance was determined as *p < 0.05, **p < 0.01 or ***p < 0.001.

## Results

### TLR4-deficient mutant C3 H/HeJ mice are more susceptible than TLR4-competent C57BL/6 J and C3 H/HeN mice to Mtb infection

To confirm the deficiency in TLR4 signaling, bone marrow-derived dendritic cells (BMDCs) differentiated from C3 H/HeN and C3 H/HeJ mice were treated with TLR2 and TLR4 agonists, respectively. BMDCs differentiation from C3 H/HeN and C3 H/HeJ mice was confirmed by flow cytometry (Fig. S1a), and more than 90% of the cells were normally differentiated into BMDCs (Fig. S1b). The LPS stimulation-induced pro-inflammatory cytokine secretion was lower in C3 H/HeJ mice than in C3 H/HeN mice (Fig. S1 c). No LPS-induced cytokine secretion was observed in BMDCs differentiated from C3 H/HeJ mice, which are TLR4 deficient. However, the secretion of TNF-α, IL-12, IL-6, IL-10, and IL-1β was observed in BMDCs differentiated from C3 H/HeJ mice, and significantly increased IL-10 was detected in BMDCs differentiated from C3 H/HeJ mice compared to BMDCs differentiated from C3 H/HeN mice (Fig. S2).

**Figure 1.**
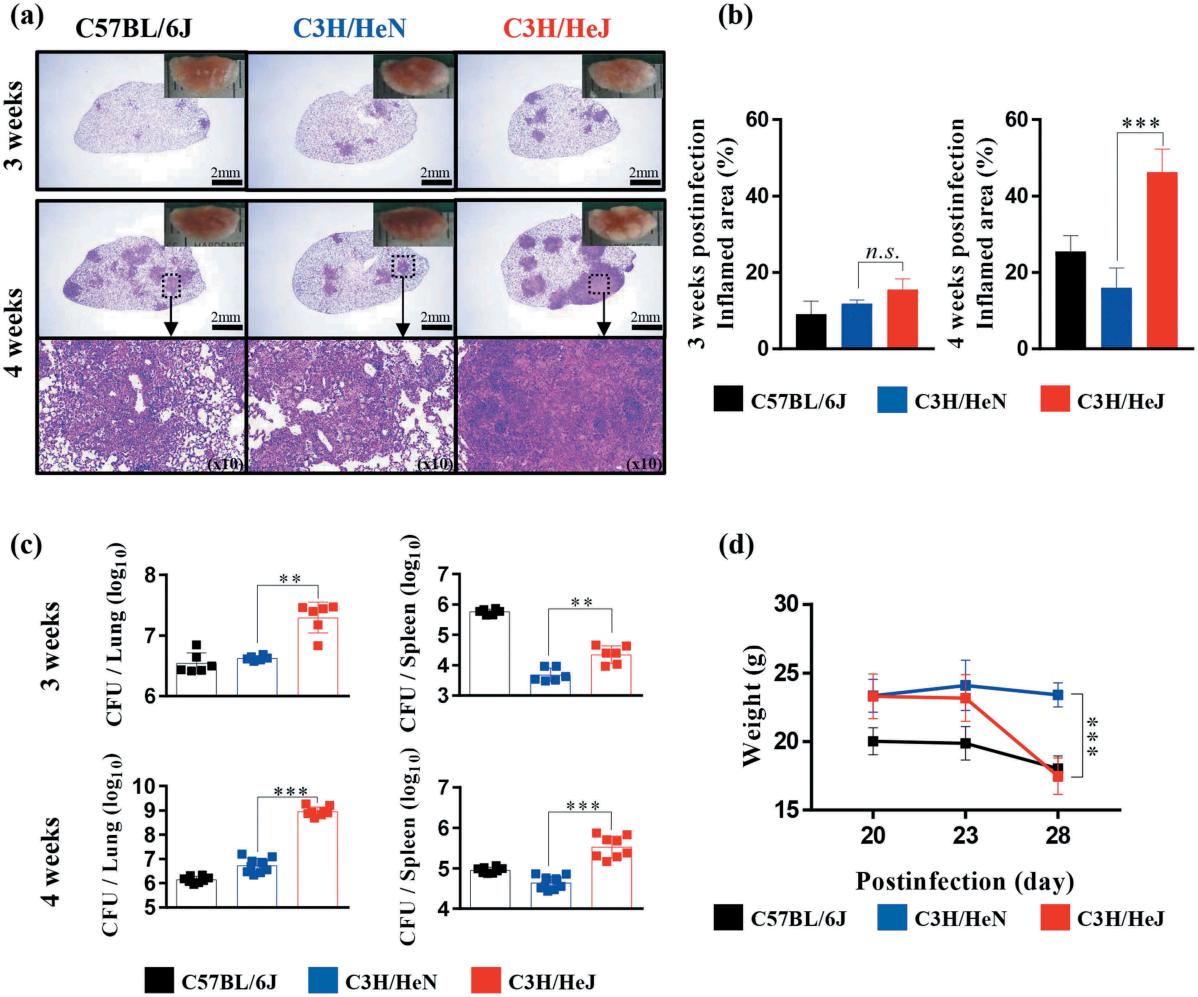
**Comparative analysis of the pathogenesis in C3 H/HeN and C3 H/HeJ mice in terms of bacterial burden and inflammation in the lungs following infection with the K strain of Mtb**. Mice were infected with 150 CFU of the Mtb K strain via aerosolization, and the lungs were removed at 3 and 4 weeks postinfection for analysis. (a) The superior lobe of the right lung stained with H&E at 3 and 4 weeks after Mtb K strain challenge. Gross lung pathology is shown with H&E staining. (b) The inflammation area of the H&E stained samples was quantified by percentage and presented as bar graphs. The data are presented as the mean ± SD of four-to-five mice in each group. The significance of differences was determined with an unpaired *t*-test. A *p* value < 0.05 was considered statistically significant. *n.s*.: not significant and ***p < 0.001. (c) The CFUs in the lungs and spleens of each group at 3 and 4 weeks postinfection were analyzed by counting the bacteria. The data are presented as the mean ± SD of four-to-five mice in each group. The significance of differences was determined with an unpaired *t*-test. A *p* value < 0.05 was considered statistically significant. **p < 0.01 and ***p < 0.001. (d) Changes in body weight were measured from 3 weeks after infection for 1 week until autopsy. The data are presented as the mean ± SD from four-to-five mice in each group. The significance of differences was determined with an unpaired *t*-test. A *p* value < 0.05 was considered statistically significant. ***p < 0.001 compared to C3 H/HeN and C3 H/HeJ.

To study the role of TLR4 during Mtb infection, we observed the pathology through H&E staining, and the bacterial burden in the lungs and spleen was analyzed at 3 and 4 weeks postinfection. The formation of granuloma structures in the lungs is a symbol of the confinement of the Mtb bacterial burden in Mtb-resistant strains of mice [26]. C57BL/6 J and C3 H/HeN mice display the typical advancement of the disease, but C3 H/HeJ mice deviated from the normal progression at 4 weeks postinfection. C3 H/HeJ mice showed a greater inflamed lung lesion width than C57BL/6 J and C3 H/HeN mice during Mtb K strain infection, which was not the case at 3 weeks postinfection (Figure 1(a,b)). The inflamed lesions in C3 H/HeJ mice were similar to the necrotic lesions in C3 HeB/FeJ mice, which are susceptible to Mtb [27]. Differences in pathology between TLR4-competent and TLR4-deficient mutant mice were evident; thus, we investigated whether dissimilarities in the bacterial burden occur during the infection phase. The bacterial burden of the Mtb K strain in the lungs of three strains was analyzed. The bacterial growth in the lungs and spleen was significantly increased in C3 H/HeJ mice compared to C57BL/6 J and C3 H/HeN mice at 4 weeks after Mtb infection (Figure 1(c)). Interestingly, the infection progression in the lungs of C3 H/HeJ mice was greatly accelerated at 4 weeks postinfection. Mtb growth was controlled in the lungs of C57BL/6 J and C3 H/HeN mice to a similar extent. The loss of weight during Mtb infection was more pronounced in C3 H/HeJ mice than C57BL/6 J and C3 H/HeN mice from 3 to 4 weeks (Figure 1(d)). These results demonstrate that TLR4 provides stronger protection against Mtb K strain infection in a mouse model.

## Lack of TLR4 signaling reduces the recruitment of T cells and promotes the influx of neutrophils into the lungs of C3 H/HeJ mice during Mtb infection

**Figure 2.**
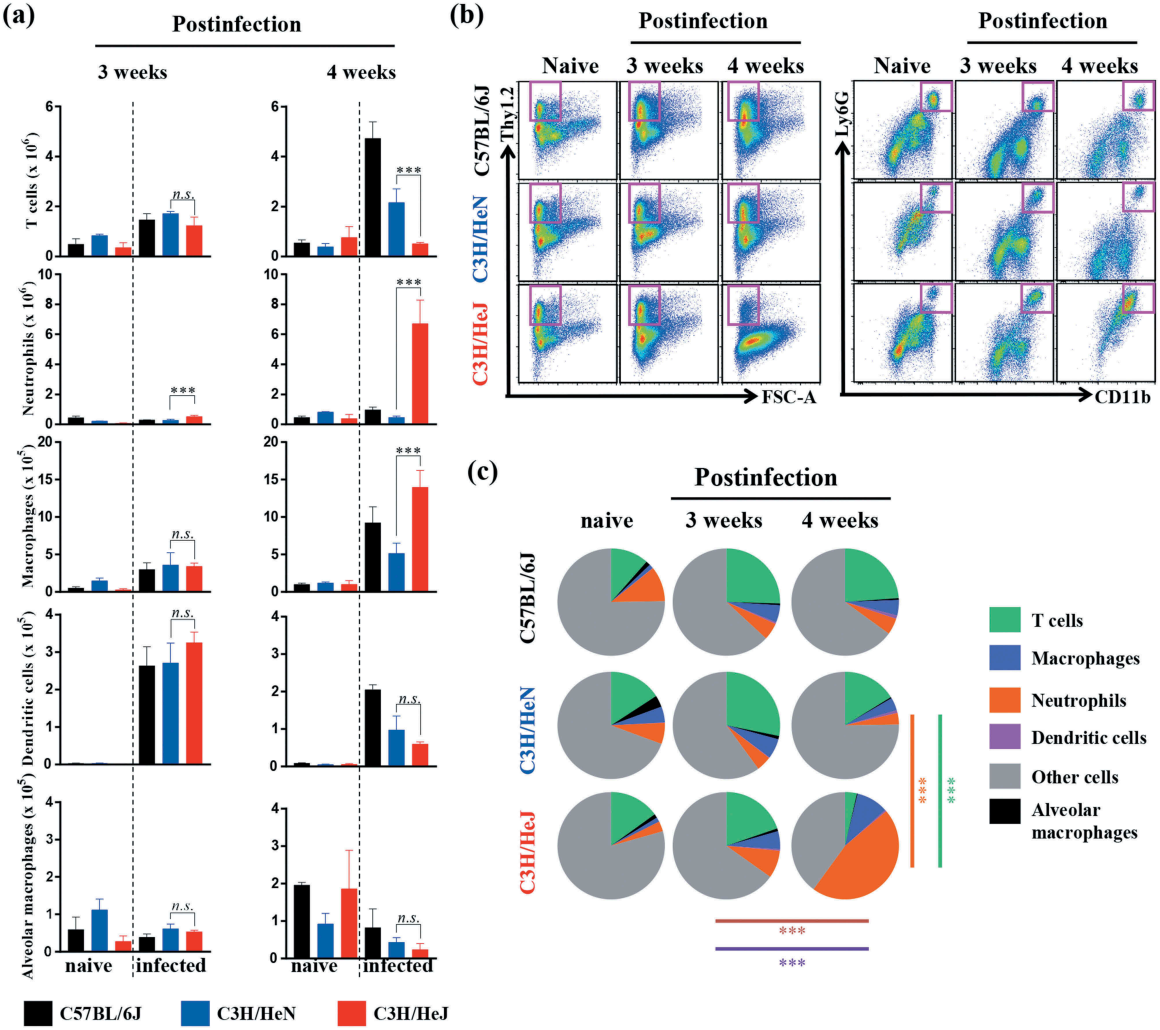
**Analysis of cellular compartments in the lungs of C57BL/6 J, C3 H/HeN and C3 H/HeJ mice after challenge with the K strain of Mtb at 3 and 4 weeks postinfection**. (a and b) The immune cells from the lungs of C57BL/6 J, C3 H/HeN and C3 H/HeJ mice were analyzed by flow cytometry, and the data are shown as bar graphs. The data are presented as the mean ± SD of four-to-five mice in each group. The significance of the differences was determined with an unpaired *t*-test. A *p* value < 0.05 was considered statistically significant; *n.s*.: not significant and ***p < 0.001. (c) Pie charts indicate the mean frequencies of the infiltrated immune cells in the lungs at 3 and 4 weeks postinfection. The data are presented as the mean ± SD of four-to-five mice in each group. The significance of the differences was determined with an unpaired *t*-test. A *p* value < 0.05 was considered statistically significant; ***p < 0.001. Orange line: significance of the neutrophil proportion between C3 H/HeN and C3 H/HeJ mice 4 weeks postinfection. Green-blue line: significance of the T cell proportion between C3 H/HeN and C3 H/HeJ mice 4 weeks postinfection. Violet line: significance of the neutrophil proportion between 3 weeks and 4 weeks postinfection in C3 H/HeJ mice. Brown line: significance of the T cell proportion between 3 weeks and 4 weeks postinfection in C3 H/HeJ mice.

We determined that the bacterial burden, inflammation and weight loss was greater in C3 H/HeJ mice than C57BL/6 J and C3 H/HeN mice due to the absence of TLR4 signaling. To identify the cell compartment that mediates the severe pathology and increased bacterial burden after infection, the influx of immune cells into the lung compartment was assessed through flow cytometry. The myeloid cells (dendritic cells, alveolar macrophages, macrophages, and neutrophils) and lymphoid cells (T cells) were examined by following a gating strategy (Fig. S3a). CD11 c^+^Siglec-F^+^ alveolar macrophages, which contribute to the first line of defense against pathogens, were not changed during Mtb infection from weeks 3 to 4 in C57BL/6 J, C3 H/HeN and C3 H/HeJ mice. The population of CD11 c^+^MHCII^+^ dendritic cells, which directly interact with activated T cells, was decreased after Mtb K strain infection at 4 weeks in C3 H/HeJ mice; however, C3 H/HeJ mice contained comparable populations at 3 weeks postinfection. The populations of other first-line defense-related immune cells, neutrophils and macrophages, were significantly increased in C3 H/HeJ mice from 3 to 4 weeks postinfection. Interestingly, the neutrophil population increased 10-fold over 3 to 4 weeks postinfection; however, the number of neutrophils in C57BL/6 J and C3 H/HeN mice tended to decrease or slightly increase (Figure 2(a)). The number of T cells, which regulate the adaptive immune response, was dramatically reduced in C3 H/HeJ mice compared to C57BL/6 J and C3 H/HeN mice at 4 weeks postinfection (Figure 2(b,c)). There was no significant difference in T cell numbers between C3 H/HeJ and C57BL/6 J, and C3 H/HeN mice at 3 weeks postinfection. These results suggest that the uncontrolled accumulation of neutrophils and insufficient number of T cells might correlate with TLR4 and Mtb susceptibility.

### Absence of TLR4 signaling leads to a decrease in the protective Th1 response and an increase in IL-10 secretion

**Figure 3.**
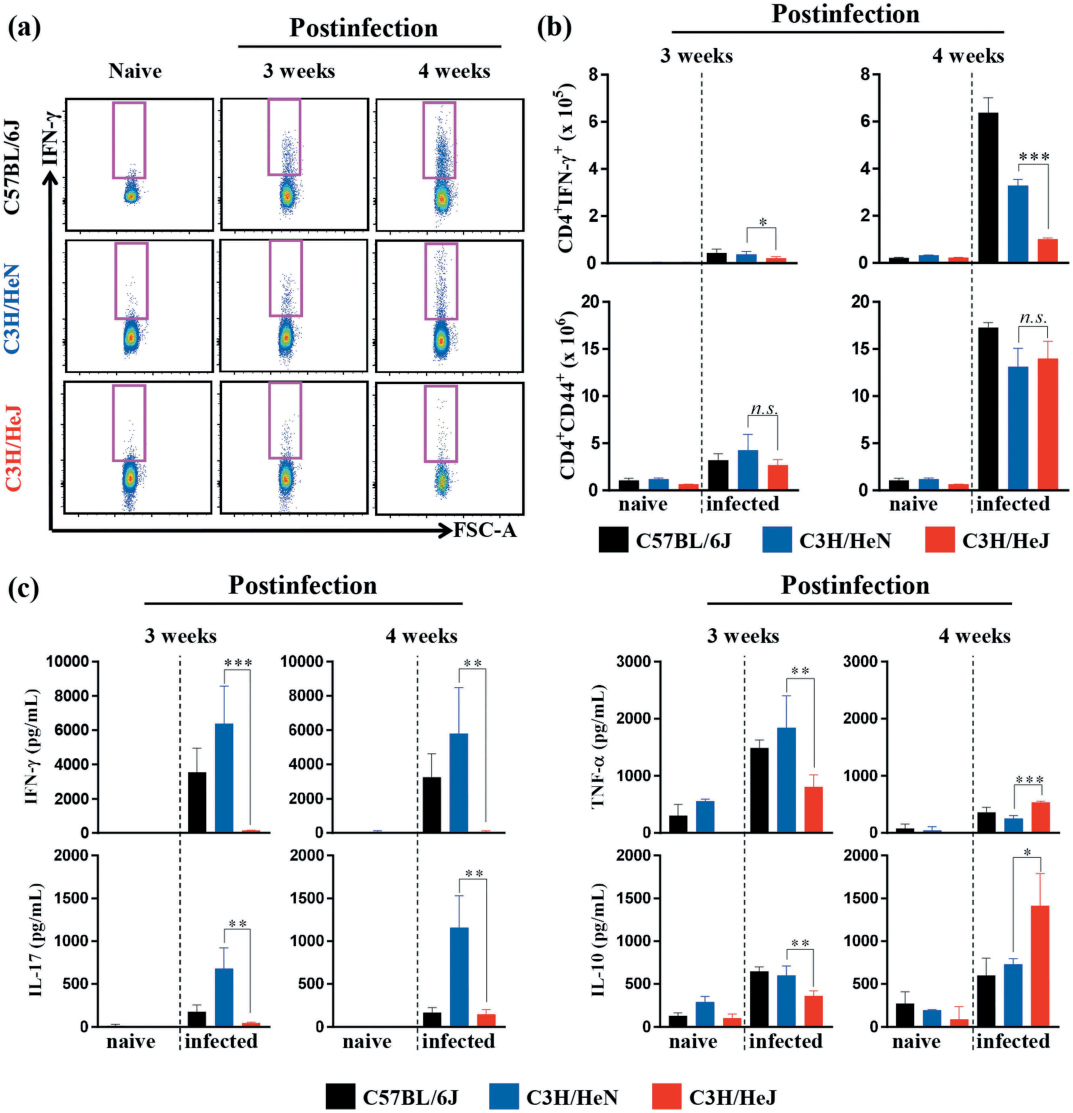
**Comparative analysis of antigen-specific T cell responses and cytokine production in the lungs of C57BL/6 J, C3 H/HeN, and C3 H/HeJ mice**. At three and four weeks postinfection, mice from each group were euthanized, and their lung cells (1 x 10^6^ cells) were stimulated with ESAT-6 (1 μg/mL) for 12 hours at 37°C in the presence of brefeldin A and monensin. (a and b) At 3 and 4 weeks postinfection, CD4^+^CD44^+^ T cells and IFN-γ-secreting CD4^+^ T cells in C57BL/6 J, C3 H/HeN and C3 H/HeJ mice were analyzed by flow cytometry, and the results are presented as bar graphs. The data are presented as the mean ± SD of four-to-five mice in each group. The significance of differences was determined with an unpaired *t*-test. A *p* value < 0.05 was considered statistically significant. *n.s*.: not significant, *p < 0.05, and ***p < 0.001. (c) The IFN-γ, IL-17AF, TNF-α, and IL-10 concentration in the supernatant were measured by ELISA. The data are presented as the mean ± SD of four-to-five mice in each group. The significance of differences was determined with an unpaired *t*-test. A *p* value < 0.05 was considered statistically significant. *p < 0.05, **p < 0.01, and ***p < 0.001.

The secretion of IFN-γ by CD4^+^ T cells is essential for reducing host damage by Mtb infection [4]. Thus, we measured the cytokine production capacity of lung cells at 3 and 4 weeks postinfection to understand the process contributing to the Mtb susceptibility of C3 H/HeJ mice. The early increase in CD4^+^IFN-γ^+^ T cells in the lungs of C57BL/6 J mice correlated with protection against Mtb infection. The number of CD4^+^IFN-γ^+^ T cells was analyzed by flow cytometry by following a gating strategy (Fig. S3b). C3 H/HeJ mice produced less IFN-γ than C57BL/6 J and C3 H/HeN mice at 3 and 4 weeks postinfection (Figure 3(a,b) top panel). However, the population of CD4^+^CD44^+^ T cells, which are activated T cells, was similar in C57BL/6 J, C3 H/HeN and C3 H/HeJ mice at 3 and 4 weeks postinfection (Figure 3(b) bottom panel). In contrast, IL-10, which is an immunosuppressive cytokine, was detected at higher concentrations in C3 H/HeJ mice than C57BL/6 J and C3 H/HeN mice at 4 weeks postinfection, in contrast to 3 weeks postinfection (Figure 3(c)). These data suggest that the Th1 response was suppressed due to the high level of the immunosuppressive cytokine IL-10, which is one of the reasons that C3 H/HeJ mice show increased susceptibility to Mtb infection.

### Neutrophil removal contributes to the control of the excessive inflammation and bacterial burden in the lungs of C3 H/HeJ mice

**Figure 4.**
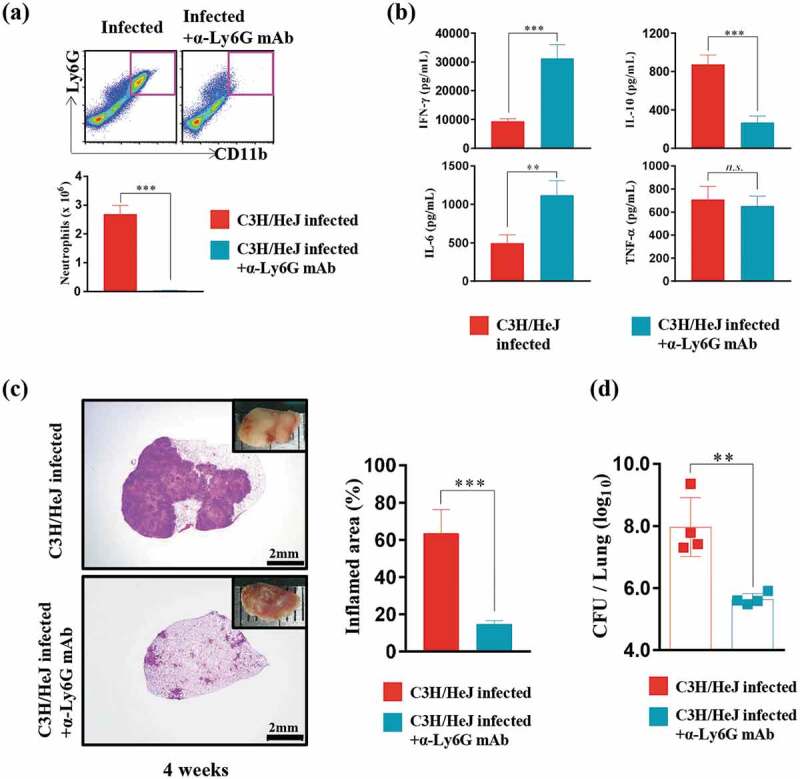
**Effect of neutrophil depletion by treatment with an anti-Ly6G monoclonal antibody on bacterial burden and inflammation in C3 H/HeJ mice**. (a) Lung cells from infected C3 H/HeJ and C3 H/HeJ mice treated with an anti-Ly6G mAb were stained with the indicated markers, and the number of neutrophils at 4 weeks postinfection is shown as a bar graph. The data are presented as the mean ± SD of four-to-five mice in each group. The significance of differences was determined with an unpaired *t*-test. A *p* value < 0.05 was considered statistically significant; ***p < 0.001. (b) At four weeks postinfection, mice from each group were euthanized, and their lung cells (1 × 10^6^ cells) were stimulated with ESAT-6 (1 μg/mL) for 12 hours at 37°C. The concentration of IFN-γ, IL-6, TNF-α, and IL-10 in the supernatant was measured by ELISA. The data are presented as the mean ± SD of four-to-five mice in each group. The significance of differences was determined with an unpaired *t*-test. A *p* value < 0.05 was considered statistically significant; *n.s*.: not significant, **p < 0.01, and ***p < 0.001. (c) The superior lobe of the right lung stained with H&E at 4 weeks after Mtb K strain challenge. The gross lung pathology is shown with H&E staining. The data represent the percentages of the superior lobe of the right lung that showed inflammation and are presented as a bar graph. The data are presented as the mean ± SD of four-to-five mice in each group. The significance of differences was determined with an unpaired *t*-test. A *p* value < 0.05 was considered statistically significant; ***p < 0.001. (d) The CFUs in the lungs of each group at 4 weeks postinfection were analyzed by counting the bacteria. The data are presented as the mean ± SD of four-to-five mice in each group. The significance of differences was determined with an unpaired *t*-test. A *p* value < 0.05 was considered statistically significant; **p < 0.01.

Compared to C3 H/HeN mice, TLR4-deficient C3 H/HeJ mice displayed a significantly increased neutrophil population. Thus, we designed an experiment to determine whether the markedly increased neutrophil population interferes with Mtb regulation in TLR4-deficient mice. The number of neutrophils in the lung was decreased after 2 weeks of Mtb infection with anti-Ly6 G mAb administration (Figure (a)). The reduction in the neutrophil number led to the recovery of the IFN-γ production and a decrease in IL-10 production (Figure 4(b)). Notably, a decrease in the inflammation area was observed after anti-Ly6 G mAb treatment in Mtb-infected C3 H/HeJ mice (Figure 4(c)). Consistent with the reduction in the size of the inflamed area, the bacterial burden decreased significantly in response to anti-Ly6 G mAb treatment after Mtb infection (Figure 4(d)).

### Blockade of IL-10 is beneficial to the host defense against Mtb infection in the absence of TLR4 signaling

**Figure 5.**
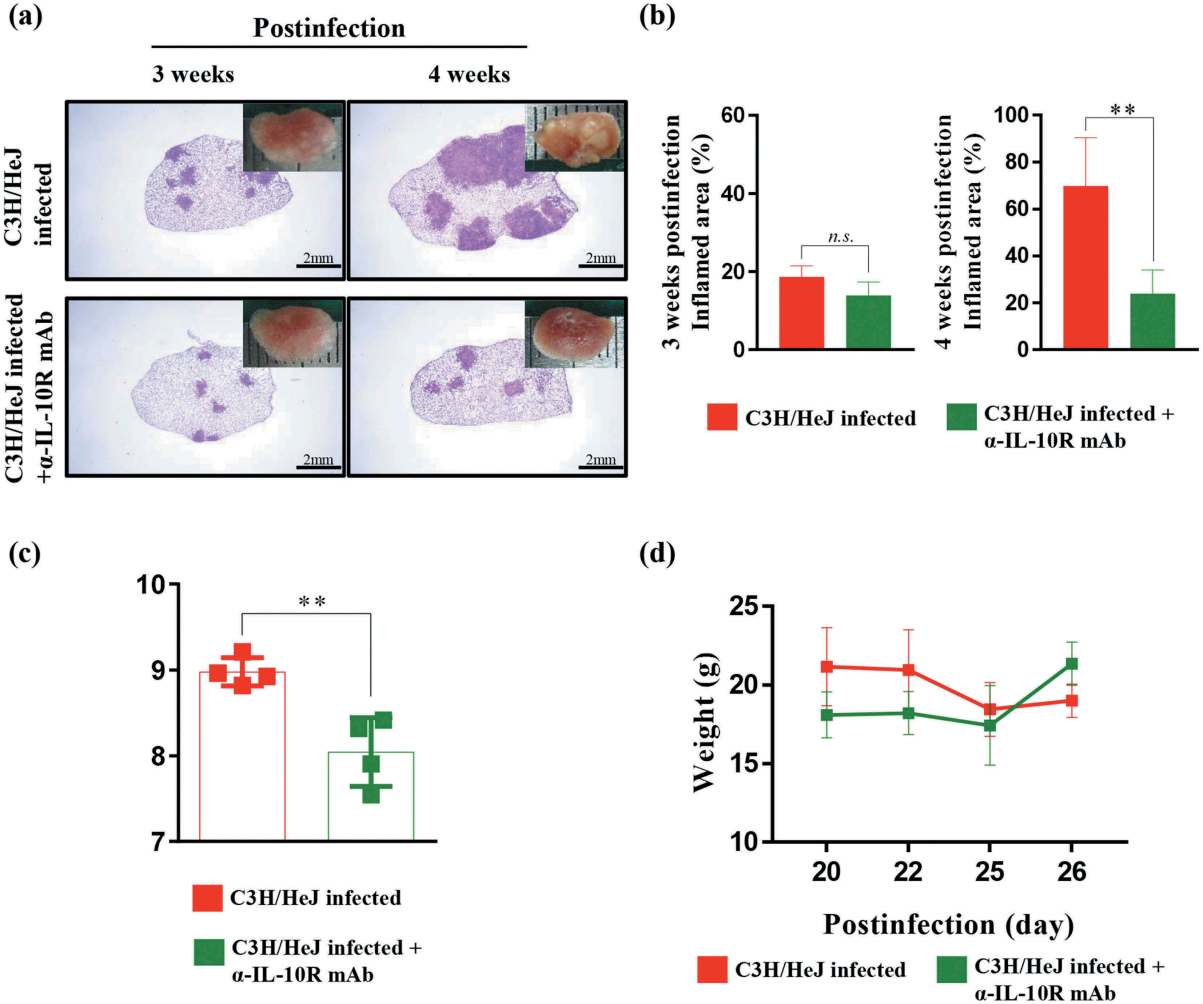
**Effect of IL-10 receptor blockade on the protection against Mtb K infection in C3 H/HeJ mice**. C3 H/HeJ and C3 H/HeJ mice intraperitoneally administered an anti-IL-10 receptor mAb weekly 2 weeks postinfection were infected with 150 CFU of the Mtb K strain via aerosolization. Subsequently, the lungs were removed at 3 and 4 weeks postinfection for analysis. (a) The superior lobe of the right lung stained with H&E at 3 and 4 weeks after Mtb K strain challenge. The gross lung pathology is shown by H&E staining. (b) The inflamed area of the H&E-stained samples was quantified as the percentage and presented in bar graphs. The data are presented as the mean ± SD of four-to-five mice in each group. The significance of differences was determined with an unpaired *t*-test. A *p* value < 0.05 was considered statistically significant; *n.s*.: not significant and **p < 0.01. (c) The CFUs in the lungs of each group at 4 weeks postinfection were analyzed by counting the bacteria. The data are presented as the mean ± SD of four-to-five mice in each group. The significance of differences was determined with an unpaired *t*-test. A *p* value < 0.05 was considered statistically significant; **p < 0.01. (d) Changes in body weights were analyzed from 20 to 27 days after Mtb K strain infection.

IL-10 is a representative cytokine that interferes with the Th1 response. As shown in Figure 3(c)), we found that IL-10 is produced in a large amount in the absence of TLR4 signaling. Thus, we hypothesized that blocking the IL-10 signal in C3 H/HeJ mice could improve the control of Mtb at 4 weeks postinfection. We treated mice with an IL-10 receptor-blocking mAb three times a week from 2 weeks to 4 weeks postinfection to reduce the damage induced by Mtb infection. The effect of the IL-10 receptor-blocking mAb was assessed by H&E staining of lung sections and gross pathology. A significant reduction in the inflammation area at 4 weeks postinfection was detected in C3 H/HeJ mice treated with the IL-10 receptor-blocking mAb (Figure 5(a,b)). The bacterial burden was significantly decreased at 4 weeks postinfection by IL-10 receptor-blocking mAb treatment (Figure 5(c)). Additionally, the weight loss was alleviated by IL-10 receptor-blocking mAb treatment (Figure 5(d)). These results indicate that the excessive secretion of IL-10 in the absence of TLR4 signaling attenuates the ability to protect against Mtb infection.

### Blockade of the IL-10 receptor restores the protective Th1 response in the lungs of C3 H/HeJ mice

**Figure 6.**
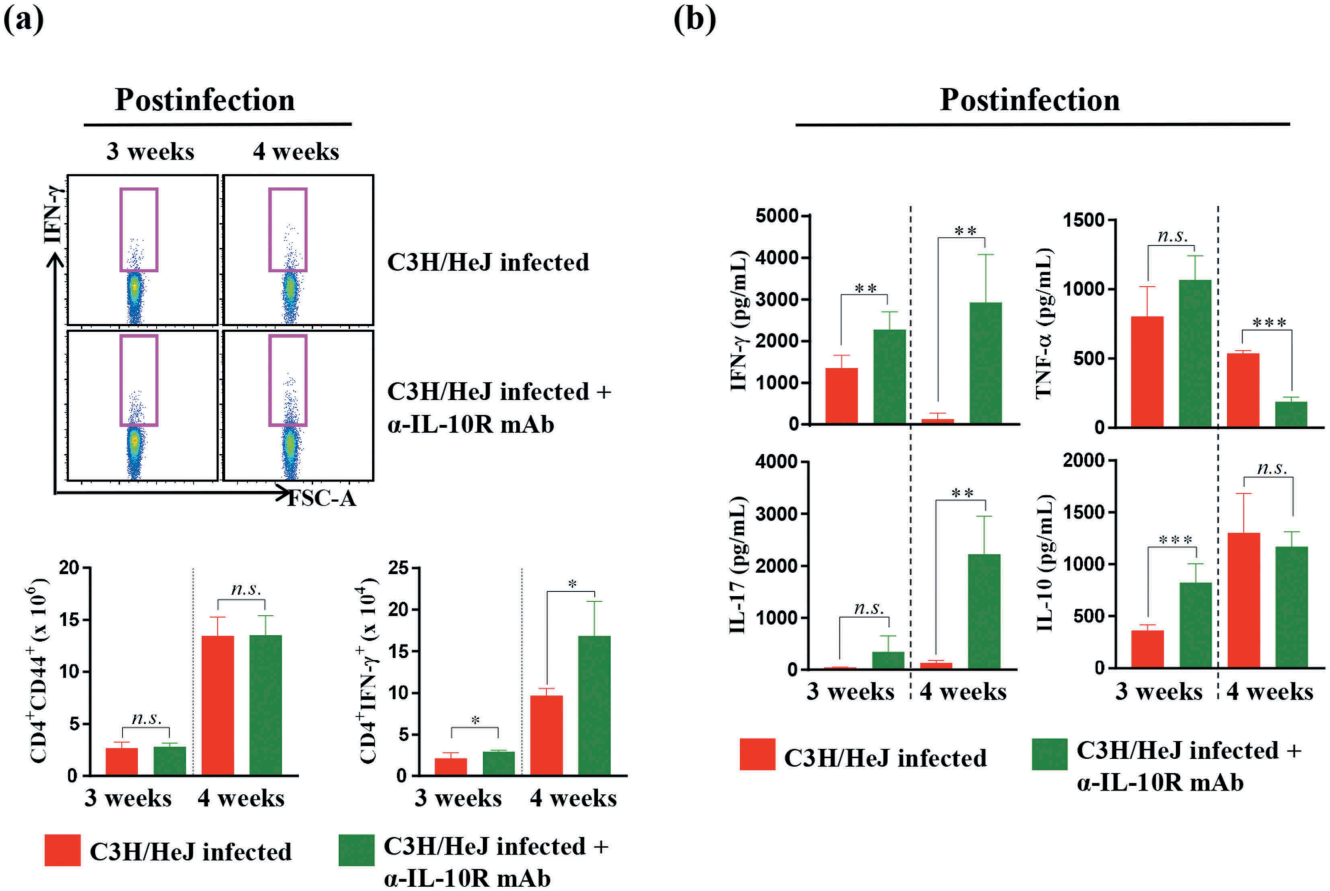
**Effect of IL-10 receptor blockade in C3 H/HeJ mice on the antigen-specific IFN-γ response and cytokine production in lung cells**. Mice from each group were euthanized, and their lung cells (1 × 10^6^ cells) were stimulated with ESAT-6 (1 μg/mL) for 12 hours at 37°C in the presence of brefeldin A and monensin. (a) At 3 and 4 weeks postinfection, the IFN-γ-secreting CD4^+^ T cells in the C57BL/6 J, C3 H/HeN and C3 H/HeJ groups of mice were analyzed by flow cytometry, and the results are presented as bar graphs. The data are presented as the mean ± SD of four-to-five mice in each group. The significance of differences was determined with an unpaired *t*-test. A *p* value < 0.05 was considered statistically significant; *n.s*.: not significant and *p < 0.05. (b) IFN-γ, IL-17AF, TNF-α, and IL-10 concentrations in the supernatants were measured by ELISA. The data are presented as the mean ± SD of four-to-five mice in each group. The significance of differences was determined with an unpaired *t*-test. A *p* value < 0.05 was considered statistically significant; *n.s*.: not significant, **p < 0.01, and ***p < 0.001.

To better understand the processes contributing to the well-controlled bacterial burden, we analyzed inflammatory cytokine secretion levels at 3 and 4 weeks postinfection. We examined the cytokine secretion of the lung cells and the number of CD4^+^IFN-γ^+^ T cells after ESAT-6 stimulation. CD4^+^IFN-γ^+^ T cells and IFN-γ production recovered, but the number of CD4^+^CD44^+^ T cells, which are activated T cells, did not differ from that in C3 H/HeJ mice at 3 and 4 weeks postinfection after IL-10 receptor-blocking mAb treatment (Figure 6(a)). The production of immunosuppressive IL-10 did not decline at 3 and 4 weeks postinfection, but the secretion of IFN-γ recovered (Figure 6(b)). These data suggest that IL-10 signaling is associated with the reduced production of IFN-γ, and IL-10 plays a role in regulating Mtb in the absence of TLR4 signaling.

### Blockade of the IL-10 receptor retains the original CD11b^hi^Ly6 G^hi^ neutrophil phenotype, whereas the CD11b^int^Ly6 G^int^ neutrophil phenotype emerges in infection-controlled C3 H/HeJ mice following Mtb infection

**Figure 7.**
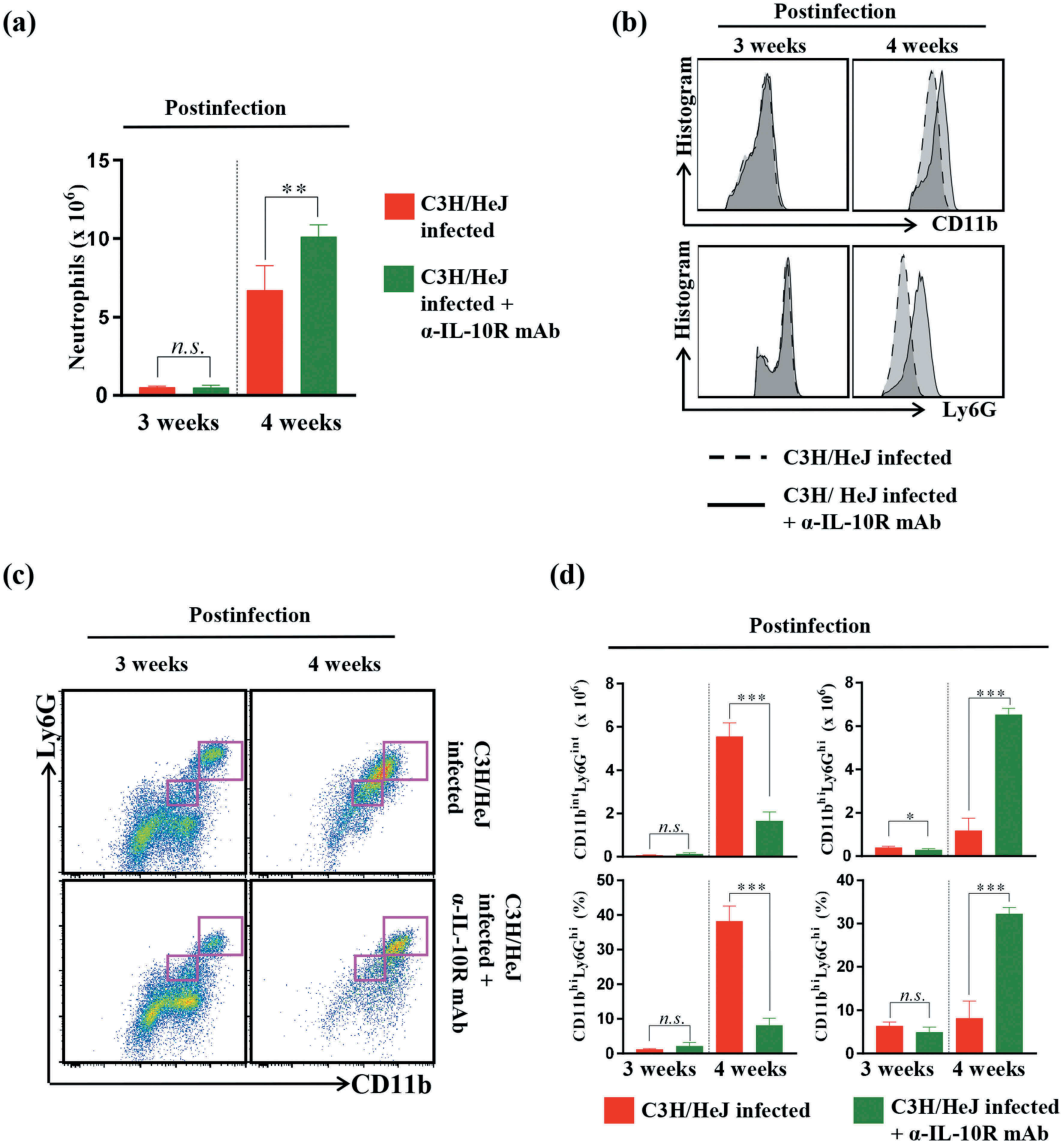
**Analysis of neutrophil phenotype alterations produced by IL-10-receptor blocking in the lungs of C3 H/HeJ mice**. The mice in each group were sacrificed at 3 and 4 weeks postinfection, and their lung cells were analyzed by flow cytometry. (a) Lung cells from infected C3 H/HeJ and C3 H/HeJ mice treated with an IL-10 receptor-blocking mAb were stained with the indicated markers, and the neutrophil populations are shown as bar graphs at 3 and 4 weeks postinfection. The data are presented as the mean ± SD of four-to-five mice in each group. The significance of differences was determined with an unpaired *t*-test. A *p* value < 0.05 was considered statistically significant; *n.s*.: not significant and **p < 0.01. (b) The expression levels of CD11b and Ly6G in the neutrophil population (CD11b^+^Ly6G^+^) were analyzed by flow cytometry and are shown as histograms. (c) Neutrophil subpopulations (CD11b^int^Ly6G^int^ and CD11b^hi^Ly6G^hi^) were gated according to the expression levels of CD11b and Ly6G. (d) Bar graph of CD11b^int^Ly6G^int^ and CD11b^hi^Ly6G^hi^ cells at 3 or 4 weeks postinfection. The data are presented as the mean ± SD of four-to-five mice in each group. The significance of differences was determined with an unpaired *t*-test. A *p* value < 0.05 was considered statistically significant; *n.s*.: not significant, *p < 0.05, and ***p < 0.001.

Next, we investigated the number of neutrophils since the blockade of the IL-10 receptor recovered the number of IFN-γ-producing CD4^+^ T cells (Figure 6), similar to the outcome of neutrophil depletion (Figure 4) in C3 H/HeJ mice. As expected, the frequency of CD4^+^ T cells was restored, but the number of CD11b^+^Ly6 G^+^ neutrophils unexpectedly increased in C3 H/HeJ mice treated with the IL-10 receptor-blocking mAb at 4 weeks after Mtb infection (Figure 7(a)). We postulated that the increased population of neutrophils in C3 H/HeJ mice treated with the IL-10 receptor-blocking mAb might display different phenotypes from those in C3 H/HeJ mice infected with Mtb only. Intriguingly, C3 H/HeJ mice treated with the IL-10 receptor blocking mAb showed a lower frequency and proportion of CD11b^int^Ly6 G^int^ cells at 4 weeks postinfection than the infection control group mice (Figure 7(b,c)). In other words, C3 H/HeJ mice mostly had CD11b^hi^Ly6 G^hi^ cells considered neutrophils at 3 weeks postinfection, but CD11b^int^Ly6 G^int^ cells emerged as neutrophils at 4 weeks postinfection. Notably, the original CD11b^hi^Ly6 G^hi^ cells that appeared at 3 weeks postinfection in C3 H/HeJ mice were retained at 4 weeks postinfection without phenotypic alteration (Figure 7(d)), suggesting that the disproportionate increase in CD11b^int^Ly6G^int^ neutrophils from CD11b^hi^Ly6G^hi^ cells may lead to the pathologic consequences in C3 H/HeJ mice.

## Discussion

In this study, we attempted to elucidate the function of TLR4 during Mtb infection because its involvement in protective immune responses against Mtb infection remains controversial. The data in our current study support the hypothesis that TLR4 is required for an optimal protective Th1 response against Mtb infection, which is in good agreement with previous reports [19,20]. In addition, we further found that Mtb infections were uncontrolled in TLR4-deficient mice due to abundant IL-10 production and an uncontrolled influx of neutrophils into the Mtb-infected lungs. The blockade of the IL-10 receptor and depletion of neutrophils restored the protective Th1 immune response against Mtb infection in the absence of TLR4. Therefore, our results clearly demonstrate that the susceptibility of TLR4-deficient C3 H/HeJ mice to Mtb infection is caused by excessive IL-10 secretion and neutrophil recruitment into the lung, resulting in the disruption of the optimal host Th1 immune response.

Recent clinical studies of TLR polymorphisms have reported their correlation with TB susceptibility in humans. In particular, the Asp299Gly mutation of *TLR4* is known to increase susceptibility to TB because of a reduced CD4^+^ T cell frequency [28]. The Asp299Gly and Thr39Ile mutations in *TLR4* are associated with increased bacillary load in the sputum and severe forms of TB in chest radiographs of TB patients [29]. Additionally, *TLR2* and *TNFA* polymorphisms along with *TLR4* polymorphisms are associated with TB infection risk [30,31]. Although these clinical data support the hypothesis that various mutations in TLR4 are closely associated with TB infection risk and TB progression, the immunological role of TLR4 in TB protection remains unclear.

Interestingly, previous studies conducted by different groups were performed using TLR4-competent C3 H/HeN and TLR4-deficient C3 H/HeJ mice, which were used in our current study. However, the outcomes between TLR4-competent C3 H/HeN and TLR4-deficient C3 H/HeJ mice infected with Mtb are clearly inconsistent among different researcher groups. Shim *et al*. and Reiling *et al*. reported that TLR4-competent C3 H/HeJ and TLR4-deficient C3 H/HeN mice infected with a low dose (50–100 CFU per mouse) of aerosolized Mtb H37Rv showed no significant differences over 100 days postinfection [21,22]. Abel *et al*. also performed experiments under conditions similar to those of these two studies. However, Abel *et al*. reported that C3 H/HeJ mice showed a more severe TB disease phenotype than C3 H/HeN mice, as demonstrated by a significantly increased bacterial load and lung inflammation [19]. One putative reason for the controversial outcomes may be the virulence of the Mtb strain. Even in the same H37Rv Mtb strain, the virulence is altered by the batch or subculture via laboratory adaption processes [32]. The pro- and anti-inflammatory cytokine secretion profile induced by TLR signaling is distinct depending on the strain and lineage of Mtb [33]. We expected that infection with Mtb K might reveal the role of TLR4 because the highly virulent Mtb K strain is associated with TB outbreaks, inducing TB progression more efficiently than the laboratory strain H37Rv [34,35]. For these reasons, in our study, infection with 150 CFU of the Mtb K strain caused severely inflamed lesions and greater bacterial burden in TLR4-deficient mice than TLR4-expressing mice (Figure 1). The second reason for the conflicting results could be the production of IL-10 and Foxp3^+^ regulatory T cells induced by hypervirulent Mtb strains, such as the HN878 strain, in the mouse model [36]. BMDMs differentiated from TLR4-deficient mutant mice produced more IL-10 after Mtb strain HN878 infection than after H37Rv infection [33]. Similarly, increased Foxp3^+^ regulatory T cell frequency and IL-10 production have been reported in TB patients [37]. Thus, IL-10 production by hypervirulent Mtb strains may be associated with the different outcomes of TLR4-competent and TLR4-deficient mice during Mtb infection. In addition, infection with a high dose of the Erdman Mtb strain by intravenous administration (1 × 10^5^ CFU), or the intranasal administration (1 × 10^5^ CFU) of Mtb strain H37Rv produced higher bacterial burdens in TLR4-deficient C3 H/HeJ mice than TLR4-competent C3 H/HeN mice, along with severe lung inflammation and decreased numbers of CD4^+^IFN-γ^+^ T cells after 2 weeks of infection [20,38]. These differences between different studies also seem to be due to the infection dose and infection route [39].

Neutrophils are important immune cells that initially control Mtb, but some studies have suggested that neutrophils promote TB progression [40,41]. In the cavity and sputum of active TB patients, neutrophils are major phagocytic cells that engulf Mtb bacilli [42]. In our study, a number of neutrophils infiltrated the lung, and a wide range of necrotic lung lesions were found in TLR4-deficient C3 H/HeJ mice (Figures 1 and 2), consistent with the results of previous studies [19,20]. In addition, the depletion of the increased neutrophils after Mtb infection significantly alleviated the bacterial burden and lesion inflammation (Figure 4). Thus, further study is necessary to investigate the functional role of infiltrated neutrophils in TLR4-deficient C3 H/HeJ mice.

Interestingly, the accumulation of neutrophils represents the disease severity and progression of TB in various TB-susceptible mouse models, including genetically susceptible mice [6,43,44]. Neutrophils and polymorphonuclear-myeloid-derived suppressor cells (PMN-MDSCs) have similar phenotypical markers, CD11b^+^Ly6G^+^ [45]. IL-10 production by MDSCs suppresses T cell responses, especially IFN-γ production by CD4^+^ T cells [46]. Recently, an increasing number of studies have reported that neutrophils are associated with the progression of TB, but the correlation of TB with MDSCs is unknown. As shown in previous studies, the removal of neutrophils in TLR4-deficient mutant mice attenuated the lesion inflammation, reduced the Mtb burden and IL-10 production, and recovered the IFN-γ production in our study. Neutrophils are known as a source of IL-10 production [47], and the depletion of neutrophils by anti-Ly6G mAb treatment was accompanied by a decrease in IL-10 production. Therefore, the increased IL-10 production through neutrophils in TLR4-deficient mice might impair the immune response against Mtb infection. The decrease in neutrophil numbers might be associated with reduction of IL-10 production because neutrophils are one of the sources of IL-10. IL-10 production via the markedly increased recruitment of neutrophils in TLR4-deficient mice could aggravate the prognosis of Mtb infection. Therefore, the removal of neutrophils could contribute to the control of Mtb infection.

The level of IL-10 was found to be elevated in the serum of TB patients with increased bacillary loads [48]. In other studies, the neutralization of endogenous IL-10 was found to elevate Th1 responses in peripheral blood mononuclear cells (PBMCs) from TB patients [49]. Previous studies have suggested that IL-10 suppresses the host protective Th1 immune response against Mtb infection, eventually leading to TB progression. In addition, similar to the results of our study, the blockade of IL-10 signaling in the Mtb-susceptible strain CBA/J has been shown to decrease the bacterial burden and restore the CD4^+^ and CD8^+^ T cell frequency [9]. The results of previous studies and our results confirm that the control of Mtb growth by IL-10 is disturbed and that the removal of IL-10 signaling enhances the protection against Mtb infection in the absence of TLR4 signaling. Furthermore, the depletion of neutrophils markedly enhanced the protection against Mtb infection and recovered the Th1-type T cell response (Figure 4). Thus, we focused on the number of neutrophils rather than other immune cells (Fig. S4). TLR4-deficient mice with blocked IL-10 signaling showed a well-controlled Mtb infection with an increased number of neutrophils (Figure 7(a)). In the current study, we found that the blockade of IL-10 signaling in TLR4-deficient mice appeared to inhibit the alteration of the conventional neutrophil phenotype, from CD11b^hi^Ly6G^hi^ to CD11b^int^Ly6G^int^ (Figure 7(b-d)). Among the cells expressing Ly6G and CD11b, Gr-1^dim^Ly6G^dim^CD11b^+^-expressing cells inhibit T cell proliferation and IFN-γ production [50]. We hypothesized that CD11b^int^Ly6G^int^ cell accumulation in TLR4-deficient mice could suppress the Th1-type T cell response. We performed an in vitro suppression assay to determine the suppressive function of CD11b^int^Ly6G^int^ cells as MDSCs after Mtb infection in the absence of TLR4 signal, but CD11b^int^Ly6G^int^ cells did not inhibit T cell proliferation (Figure S5). Although CD11b^int^Ly6G^int^ cells did not have the function of MDSCs [51], this cell population may be involved in pathogenesis through other mechanisms that need to be further studied. In addition, the phenotypic maintenance of neutrophils caused by the blockade of the IL-10 receptor may contribute to the restriction of the progression of TB. An increase in granulocyte colony-stimulating factor (G-CSF) was reported to cause neutrophil accumulation in TB patients [52,53]. These reports suggested a possibility that the increase of neutrophils is caused by growth factors such as G-CSF after Mtb infection, but further research is required to determine whether TLR4 is a directly related to the secretion of G-CSF.

In summary, our results suggest that TLR4 is necessary to elicit optimal protection against Mtb infection via the regulation of IFN-γ, IL-10 production, and neutrophil recruitment during Mtb infection. The disruption of IL-10 signaling and the depletion of neutrophils contribute to restrict Mtb growth in TLR4-deficient mice and enhance the IFN-γ-producing CD4^+^ T cell response. Although the alteration of neutrophil phenotypes by IL-10 was associated with TB progression, it did not affect the function after Mtb infection. These findings could provide effective targets and immunological approaches for understanding TB pathogenesis.
